# Amitriptyline Induced Life‐Threatening Steven–Johnson Syndrome: Case Report

**DOI:** 10.1002/ccr3.72129

**Published:** 2026-02-22

**Authors:** Wondwosen Mengist Dereje, Lidya Yalew, Nardos Tafesse, Teklegiorgis Goshime Getachew

**Affiliations:** ^1^ Department of Neurology University of Gondar College of Medicine and Health Sciences Gondar Ethiopia; ^2^ Department of Internal Medicine University of Gondar College of Medicine and Health Sciences Gondar Ethiopia; ^3^ Department of Emergency and Critical Care Medicine University of Gondar College of Medicine and Health Sciences Gondar Ethiopia

**Keywords:** adverse drug reaction, amitriptyline, case report, Stevens–Johnson syndrome

## Abstract

Stevens–Johnson syndrome is one of the few dermatological emergencies in clinical practice. The syndrome is often secondary to the usage of drugs, of which allopurinol, penicillins, sulfa drugs, ibuprofen, sodium valproate, phenytoin, lamotrigine, and carbamazepine are commonly implicated. Stevens–Johnson syndrome due to amitriptyline combination has not been reported in the literature before.

## 1 Introduction

Drug reactions are undesired bodily responses that occur following the administration of medications and are not characteristic of their intended pharmacodynamic effects [1]. These reactions range from transient erythema to severe manifestations collectively referred to as severe cutaneous adverse reactions (SCAR) [2].

Stevens–Johnson syndrome (SJS) is a rare but potentially life‐threatening condition, almost always triggered by medications. The average annual incidence in the United States is estimated to be approximately 1.2 to 6 cases per million, with a male‐to‐female ratio of 1.5:1. However, a study conducted by Patel TK et al. found no significant differences in incidence based on age or sex within the Indian population [3].

SJS is more likely to occur in high‐risk groups such as slow acetylators, immunocompromised individuals, and patients with specific HLA associations [4]. Individuals carrying the HLA‐B*1502 allele are at increased risk of developing carbamazepine‐induced SJS. The US Food and Drug Administration (US‐FDA) has issued a labeling change for carbamazepine to reflect this association [3]. This allele is found predominantly among Chinese and Korean populations [5].

Similar genetic associations have been identified for other drugs, including abacavir (HLA‐B*5701*) *and allopurinol* (*HLA‐B*5801) [6].

In the reported case, HLA typing was not performed because the required analytical equipment was unavailable.

## 2 Case History

A 55‐year‐old female patient was referred from a primary hospital to our tertiary care center for social reasons and further evaluation after being admitted at the primary hospital for 1 week. Initially, she presented there with a complaint of multiple erythematous skin rashes of 3 days' duration and rapidly progressive shortness of breath that began 2 h prior to presentation.

She had been diagnosed with trigeminal neuralgia 6 years earlier and had been taking carbamazepine consistently during that time, with no previous similar illness. Three weeks prior to the onset of the rash, she visited her follow‐up clinic because her pain was no longer adequately controlled with carbamazepine alone. Consequently, she was prescribed amitriptyline 25 mg orally once daily as an adjunct therapy.

After initiating combination therapy with carbamazepine and amitriptyline, her pain subsided, and she was able to resume her routine daily activities. However, 3 weeks after starting amitriptyline, she developed a diffuse erythematous skin rash that began with itching on her back. She initially did not notice the rash, as she reported having no mirrors in the house, and attributed the itching to irritation from the jar she carried while fetching water. When the itching persisted, she asked her daughter to examine her back and was found to have a diffuse rash, which subsequently appeared on her face and spread to involve the entire body within 3 days.

The rash was associated with pain and itching. On the third day of the rash, she began to experience shortness of breath, which rapidly worsened to the point where she was unable to breathe. She was rushed to the hospital, where she lost consciousness upon arrival.

At the primary hospital, she was immediately transferred to the intensive care unit (ICU) and intubated. Based on the initial assessment, a diagnosis of life‐threatening Stevens–Johnson syndrome was made. She was started on intravenous hydrocortisone 100 mg four times daily, intravenous fluids with Ringer's lactate, and paracetamol for symptomatic relief.

All the suspected offending drugs were discontinued. After 24 h of intubation, the patient regained full consciousness and was successfully extubated. On the fourth day, hydrocortisone was switched to oral prednisolone 50 mg daily, and the skin rash gradually became less prominent.

Following 1 week of management, she was referred to our tertiary center for further investigation, advanced management, and social support.

Upon presentation to our facility, she appeared acutely ill, with visible rashes over her face. Her vital signs were as follows: blood pressure 120/70 mmHg, pulse rate 96 beats per minute, respiratory rate 17 breaths per minute, temperature 37.1°C, and oxygen saturation 97% on room air.

**FIGURE 1:**
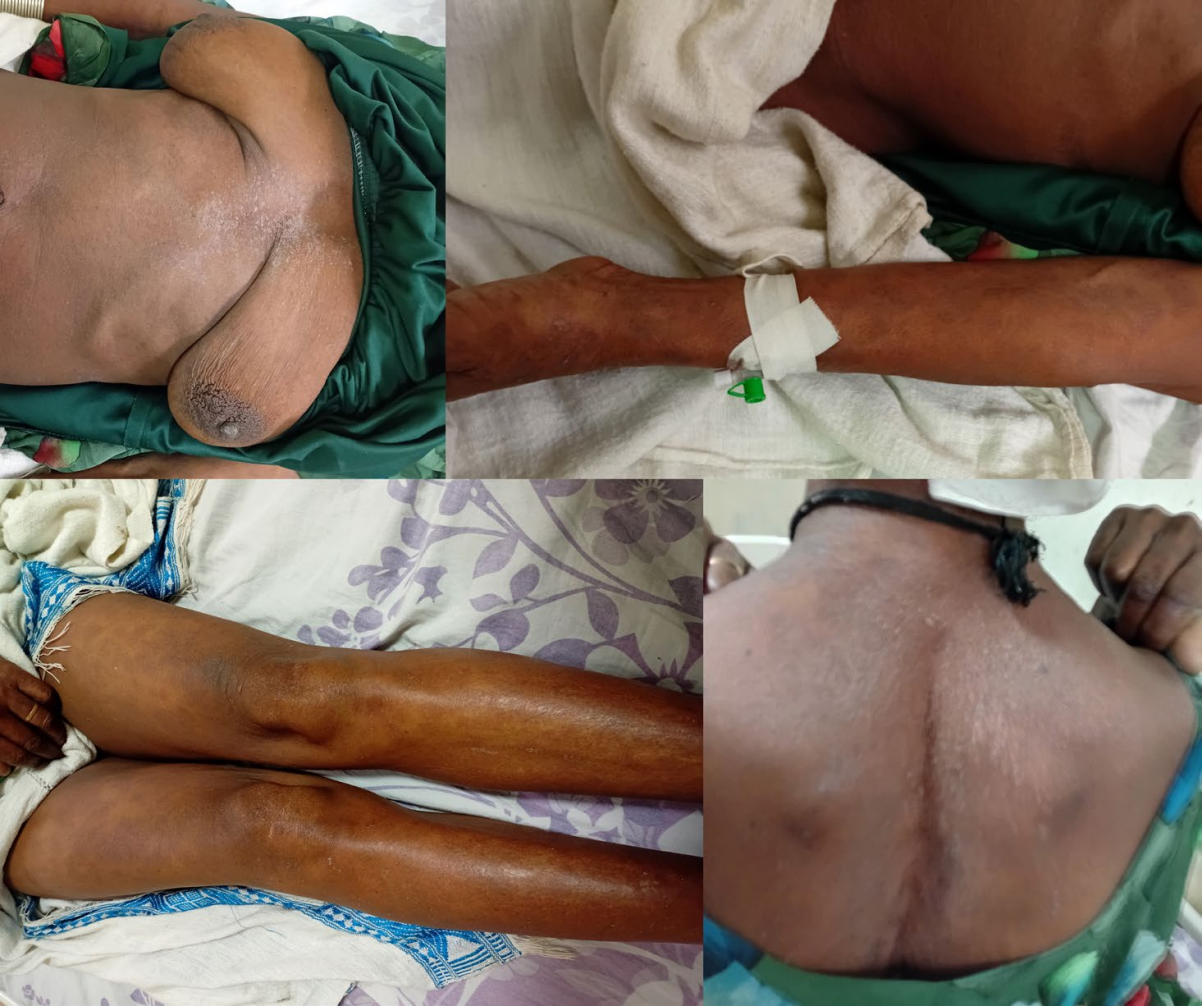
Diffuse healing macular lesions over different parts of the patient's body.

**FIGURE 2:**
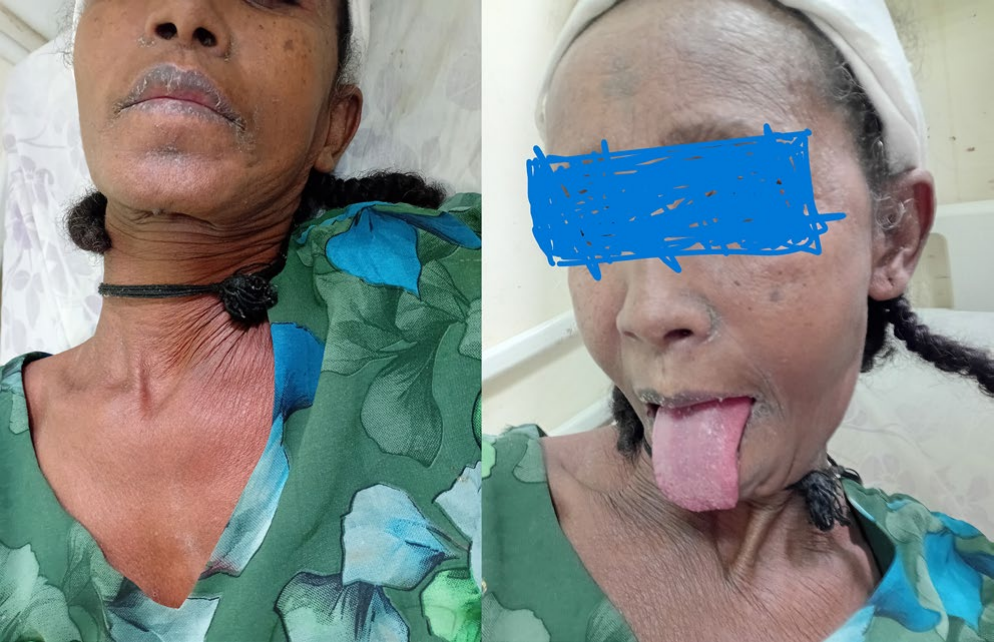
Mucosal involvement including the tongue.

Her random blood glucose level was 103 mg/dL. On physical examination, chest examination revealed clear lung fields with good bilateral air entry. Examination of the integumentary system showed a diffuse macular rash covering her entire body (Figure 1). There are also lesions over the buccal area including the tongue (Figure 2) along with involvement of the urogenital mucosa.

Other systemic examinations were unremarkable.

## 3 Conclusion and Results

A dermatology consultation was obtained, and after thorough evaluation, the team considered amitriptyline to be the likely cause of the skin eruption. They advised discontinuation of the drug and recommended cautious reconsideration of carbamazepine only after complete symptom resolution.

Initially, both medications were discontinued, and the patient was discharged on gabapentin 300 mg orally twice daily. Her symptoms subsequently resolved.

At follow‐up visits after 2, 4, and 8 weeks in the neurology outpatient clinic, the skin rash had completely subsided, and her neuralgic pain was well controlled with gabapentin. She continued her regular follow‐up at the neurology clinic.

## 4 Discussion

More than one hundred drugs have been reported to cause Stevens–Johnson syndrome (SJS) and toxic epidermal necrolysis (TEN) [7]. Commonly implicated agents include nonsteroidal anti‐inflammatory drugs (NSAIDs), allopurinol, antiepileptics, and antibiotics [4, 8, 9].

Among antiepileptic drugs, cases of SJS have been documented following the use of phenytoin, carbamazepine, valproic acid, lamotrigine, and levetiracetam, among others [10, 11]. In a study conducted by Sasidharanpillai et al., phenytoin was identified as the most frequently implicated drug, followed by carbamazepine [12].

According to age‐based analysis, the most commonly associated drugs in SJS/TEN, along with their incidence rates, are presented in Table 1 [7].

**TABLE 1:** Incidence of SJS/TEN with the most commonly implicated drugs.

Adults	Children
Nevirapine (1 in 1000)	Nevirapine (3 in 1000)
Lamotrigine (1 in 1000)	Lamotrigine (3 in 1000)
Carbamazepine (14 in 100,000)	Sulfonamides
Sulfadoxine‐Pyrimethamine (10 in 100,000)	Acetaminophen
Cotrimoxazole (1 to 3 in 100,000)	Other anti‐epileptics

European data suggest that allopurinol may be the most frequently implicated drug on the continent [7]. Seasonal variation has also been observed, particularly with cotrimoxazole, which tends to occur more commonly during the spring season [13]. However, similar patterns have not been demonstrated for other causative drugs.

In a study conducted by Vertieva et al. in Russia, the highest severity of SJS was associated with sulfamethoxazole, methimazole, and carbamazepine [14]. A review of the literature indicates that the incidence of SJS/TEN is highest within the first 2 months of initiating the offending medication, with a marked decline thereafter [15].

In the reported case, the patient had been taking carbamazepine for 6 years without any prior similar manifestations. However, after taking amitriptyline for 3 weeks, she developed Stevens–Johnson syndrome.

Although it was not possible to definitively determine whether carbamazepine or amitriptyline was responsible, the timing of symptom onset suggests that amitriptyline was the more likely causative agent.

To the best of our knowledge, this is the first reported case of SJS associated with amitriptyline.

The most common clinical features of SJS include fever, flu‐like symptoms, and skin eruptions that initially appear as macular lesions and later evolve into bullous, target‐like lesions. Involvement of the mucous membranes may also occur, although it is not present in all patients. The diagnosis is typically established based on the characteristic clinical presentation and confirmed by skin biopsy [7].

Skin lesions typically first appear on the trunk and then spread to the neck, face, and proximal portions of the upper extremities. The distal parts of the arms and legs are generally spared, although the palms and soles may be among the earliest sites of involvement. Erythema and erosions of the buccal, ocular, and genital mucosa occur in more than 90% of patients. Involvement of the respiratory tract epithelium is seen in approximately 25% of cases of toxic epidermal necrolysis (TEN), and gastrointestinal lesions may also develop. The cutaneous lesions are usually tender, while mucosal erosions are notably painful [16, 17].

In our case, the patient exhibited involvement of both the buccal and urogenital mucosa.

In the reported case, the patient presented to our facility after 1 week of steroid use, by which time the lesions had already subsided. However, based on the patient's description, the dermatologic manifestations were typical of Stevens–Johnson syndrome. Additionally, the improvement of the lesions following discontinuation of the offending medications supports the diagnosis.

Although the patient presented to our facility 1 week after symptom onset, we could not directly observe the initial lesions; we had the referral documentation from the primary hospital and communicated with the treating physicians there to confirm the patient's clinical presentation.

What makes this case unusual is the presence of severe mucosal involvement, which progressed to the point of airway compromise.

The SCORTEN scale, introduced in 2000, is the most commonly used tool to predict mortality in patients with SJS/TEN. This scoring system considers factors such as patient age, extent of skin lesions, tachycardia, presence of malignancy, metabolic abnormalities, and renal failure [6, 7].

In the reported case, the patient had a SCORTEN score of 2, corresponding to a predicted mortality rate of 12.1%. Fortunately, the patient survived.

Severe cutaneous adverse drug reactions have been described, including serum sickness–like reaction, acute generalized exanthematous pustulosis (AGEP), drug reaction with eosinophilia and systemic symptoms (DRESS), and epidermal necrolysis [18]. Epidermal necrolysis–spectrum eruptions, such as Stevens–Johnson syndrome (SJS) and toxic epidermal necrolysis (TEN), are considered a continuum and share the same pathogenesis [19].

Serum sickness (SS) is a Type III hypersensitivity reaction characterized by the deposition of immune complexes, which leads to activation of immune cells expressing Fc receptors [20].

Multiple studies have identified β‐lactams—primarily amoxicillin and cephalosporins—and sulfonamides as the antibiotics most commonly associated with serum sickness–like reaction (SSLR) [21]. The typical symptoms of SSLR include fever, arthralgia, and a maculopapular rash, usually appearing 1–2 weeks after starting the medication [22].

In the reported case, the skin rash appeared 3 weeks after initiating amitriptyline, making SSLR unlikely. In patients with prior exposure to the causative agent, the reaction typically occurs more rapidly [23], further suggesting that carbamazepine was unlikely to be the culprit.

Acute generalized exanthematous pustulosis (AGEP) is a rare, severe, acute cutaneous adverse reaction, most commonly triggered by drugs, although other causes have also been reported [24]. It is characterized by edematous erythema, often affecting large skin folds, followed by the appearance of numerous small, nonfollicular, sterile pustules and subsequent typical desquamation [25].

The clinical course of AGEP is notable for its sudden onset [26], typically occurring within 24–48 h of exposure to the offending drug, with a median onset of 24 h [27]. Given the patient's clinical presentation and the timing of symptom onset, AGEP is considered an unlikely diagnosis in this case.

Drug reaction with eosinophilia and systemic symptoms (DRESS) is a severe T‐cell–mediated cutaneous adverse reaction, characterized by rash, fever, internal organ involvement, and systemic manifestations after prolonged exposure to a medication. Patients often experience a prodromal phase with fever, malaise, sore throat, dysphagia, pruritus, or a burning cutaneous sensation, followed by a morbilliform eruption that typically begins on the trunk and face and may eventually involve over 50% of the body surface area [28]. Facial edema, which can accentuate or create new oblique earlobe creases, is a characteristic feature that helps distinguish DRESS from uncomplicated morbilliform drug eruptions [29].

The clinical presentation of our patient does not correspond to these features, making DRESS an unlikely diagnosis.

Stevens–Johnson syndrome (SJS), SJS/TEN overlap, and toxic epidermal necrolysis (TEN) represent a continuum of the same condition, classified according to the percentage of body surface area (BSA) involved: < 10% for SJS, 10%–30% for SJS/TEN overlap, and > 30% for TEN [21]. In the reported case, the diagnosis was made as SJS because the total BSA affected was less than 10%.

Amitriptyline‐induced skin necrosis could also be considered as a differential diagnosis; however, the presence of significant mucosal involvement makes this diagnosis unlikely.

Management of SJS/TEN typically resembles that of burn patients. Fluid and metabolic corrections are essential, although the deficits are generally less severe than those observed in burns. Optimal care involves admission to a dedicated burn unit or an intensive care unit (ICU); however, the risk of infection spread is significantly higher in the latter setting [7].

The initial step in management is the immediate withdrawal of the offending drug, which was implemented in this case. This is followed by administration of corticosteroids, either intravenously or orally, and, in severe cases, intravenous immunoglobulins may be considered [12, 30].

The use of systemic corticosteroids in SJS/TEN remains controversial for several reasons:

- Increased risk of infections.
- Potential to mask sepsis.
- Delayed skin epithelialization.
- Lack of controlled trials demonstrating clear benefit.

Nevertheless, substantial evidence suggests that corticosteroids can prevent or delay ocular complications associated with the disease. Therefore, their use is typically guided by the clinical judgment of the treating physician and the patient's condition [7, 31].

In addition to supportive care and wound management, immunosuppressive therapy is considered potentially beneficial in SJS/TEN due to the immunologic nature of the disease. Corticosteroids suppress inflammatory and immune responses and have served as the first‐line treatment for SJS/TEN for over 30 years [32].

In the reported case, the patient presented with life‐threatening airway obstruction, and the administration of corticosteroids was an appropriate decision given the immediate risk to life.

Although intravenous immunoglobulin (IVIG) is also used in the management of SJS/TEN, the largest trial to date, which included 281 patients, demonstrated no significant benefit. Therefore, IVIG was not considered in the treatment of our patient [33].

In cases involving extensive desquamation of the skin and mucosa, procedures such as skin grafting and amniotic membrane ocular transplantation may be performed [34]. In our patient, such interventions were not necessary.

An important aspect of patient management also includes reporting the adverse drug reaction through an appropriate local pharmacovigilance program [35].

## 5 Limitation

Although this case represents an extremely rare adverse drug reaction to amitriptyline, the diagnosis was based on the classic clinical presentation, appropriate response to standard treatment for Stevens–Johnson syndrome, and complete clinical improvement following discontinuation of the suspected drug. Although histopathological evaluation would have been valuable for diagnostic confirmation, the patient declined a skin biopsy, and therefore it could not be performed. Nevertheless, considering the overall clinical features and therapeutic response, the diagnosis was considered most consistent with amitriptyline‐induced disease, with amitriptyline regarded as the most likely causative agent.

Although HLA typing would have been valuable for this patient, it could not be performed due to limitations in the available resources.

## Author Contributions

**Wondwosen Mengist Dereje:** conceptualization, data curation, formal analysis, validation, visualization, writing – original draft, writing – review and editing. **Lidya Yalew:** visualization, writing – original draft, writing – review and editing. **Nardos Tafesse:** visualization, writing – original draft, writing – review and editing. **Teklegiorgis Goshime Getachew:** supervision, validation, visualization, writing – original draft, writing – review and editing.

## Funding

The authors have nothing to report.

## Ethics Statement

Ethical approval for this study was provided by the Ethical Committee of our institution at Gondar university hospital, Gondar, Ethiopia on September 2025.

## Consent

Written informed consent was obtained from the patient for the publication of this case report and any accompanying images. A copy of the written consent is available for review by the editor in chief of this journal on request.

## Conflicts of Interest

The authors declare no conflicts of interest.

## Data Availability

Due to patient privacy concerns, we are unable to share the data publicly. However, the data can be made available to the Editor‐in‐Chief upon reasonable request through the corresponding author.
